# Functionally confirmed compound heterozygous *ADAM17* missense loss-of-function variants cause neonatal inflammatory skin and bowel disease 1

**DOI:** 10.1038/s41598-021-89063-0

**Published:** 2021-05-05

**Authors:** Issei Imoto, Masako Saito, Kenichi Suga, Tomohiro Kohmoto, Masanobu Otsu, Keisuke Horiuchi, Hironao Nakayama, Shigeki Higashiyama, Mayumi Sugimoto, Ayumi Sasaki, Yukako Homma, Miki Shono, Ryuji Nakagawa, Yasunobu Hayabuchi, Shoichiro Tange, Shoji Kagami, Kiyoshi Masuda

**Affiliations:** 1 Risk Assessment Center, Aichi Cancer Center Hospital, Nagoya, Japan; 2 Division of Molecular Genetics, Aichi Cancer Center Research Institute, Nagoya, Japan; 3 Department of Human Genetics, Graduate School of Biomedical Sciences, Tokushima University, Tokushima, Japan; 4 Department of Pediatrics, Graduate School of Biomedical Sciences, Tokushima University, Tokushima, Japan; 5 Department of Dermatology, Graduate School of Biomedical Sciences, Tokushima University, Tokushima, Japan; 6 National Defense Medical Collage, Saitama, Japan; 7 Department of Biochemistry and Molecular Genetics, Ehime University Graduate School of Medicine, Ehime, 791-0925 Japan; 8 Kawasaki Medical School, Kurashiki, 701-0192 Japan

**Keywords:** Biochemistry, Genetics, Diseases, Molecular medicine

## Abstract

A disintegrin and metalloprotease 17 (ADAM17) is the major sheddase that processes more than 80 substrates, including tumour necrosis factor-α (TNFα). The homozygous genetic deficiency of *ADAM17* causing a complete loss of ADAM17 expression was reported to be linked to neonatal inflammatory skin and bowel disease 1 (NISBD1). Here we report for the first time, a family with NISBD1 caused by functionally confirmed compound heterozygous missense variants of *ADAM17*, namely c.1699T>C (p.Cys567Arg) and c.1799G>A (p.Cys600Tyr). Both variants were detected in two siblings with clinical features of NISBD1, such as erythroderma with exudate in whole body, recurrent skin infection and sepsis and prolonged diarrhoea*.* In a cell-based assay using *Adam10/17* double-knockout mouse embryonic fibroblasts (*Adam10*/*17*^−/−^ mEFs) exogenously expressing each of these mutants, phorbol 12-myristate 13-acetate-stimulated shedding was strongly reduced compared with wild-type ADAM17. Thus, in vitro functional assays demonstrated that both missense variants cause the loss-of-function of ADAM17, resulting in the development of NISBD1. Our study further expands the spectrum of genetic pathology underlying ADAM17 in NISBD1 and establishes functional assay systems for its missense variants.

## Introduction

A disintegrin and metalloproteinase 17 (ADAM17), also known as tumour necrosis factor α (TNFα)-converting enzyme (TACE), is a membrane-bound shedding protease, which cleaves more than 80 substrates ranging from cytokines, growth factors and receptors to cell adhesion molecules^1,2^. ADAM17 is essential for development through shedding various growth factors, e.g. the epidermal growth factor receptor (EGF-R) ligands including transforming growth factor α (TGFα), amphiregulin, epiregulin and heparin-binding (HB)-EGF; ADAM17 knockout leads to perinatal lethality in mice^1–4^. ADAM17 also plays a decisive role in inflammation through shedding cytokines and cytokine receptors including TNFα, the TNF receptors 1 and 2 (TNF-R1/2) and interleukin-6 receptor (IL-6R)^2^. Owing to its large substrate profile, ADAM17 is involved in various pathological conditions, including cancer, inflammation, neurodegeneration and fibrosis^2^. Loss-of-function variants of *ADAM17* were identified in 4 patients, two of whom are siblings, from three families with autosomal recessive neonatal inflammatory skin and bowel disease 1 (NISBD1, OMIM # 614328), which is characterised by inflammatory features with neonatal-onset, involving the skin, hair and gut (Table 1)^5–7^. In NISBD1, skin lesions consist of perioral and perianal erythema with fissuring and a generalized pustular rash that may develop into psoriasiform erythroderma. The skin disease seems to undergo phases of exacerbation and remission, with recurrent flares of erythema, scaling and widespread pustules. Gastrointestinal symptoms include malabsorptive diarrhoea, which is exacerbated by intercurrent gastrointestinal infections. The hair is short or broken, and eyelashes and eyebrows are wiry and disorganised.

**Table 1:** Clinical features of patients molecularly diagnosed with NISBD1 in presented and reported cases.

	Present case 1	Present case 2	Case 1	Case 2	Case 3	Case 4
Sex	Male	Male	Male	Female	Female	Male
Perinatal history (birth weight)	32 w of GA (1144 g)	37 w of GA (2066 g)	Term	Term	Term (2790 g)	37w of GA (2185 g)
Consanguinity of parents	No	No	Yes (first cousins)	Yes (first cousins)	No	Yes (first cousins)
Erythroderma	+	+	+	+	+	+
Hair	Alopecia	Alopecia	Short/broken	Short/broken	Present at birth but shed soon thereafter	Alopecia
Eyelashes and eyebrows	Present at birth but shed soon thereafter	Present at birth but shed soon thereafter	Wiry/ disorganised	Wiry/ disorganised	Present at birth but shed soon thereafter	sparse
Refractory diarrhoea	+	+	+	+	+	+
Recurrent infection	+ Multiple (e.g. skin, sepsis)	+ Multiple (e.g. skin, sepsis)	+ Multiple (e.g. Blepharitis, Otitis externa)	+ Multiple (e.g. blepharitis, otitis externa)	+ Multiple (e.g. skin, sepsis)	+ Multiple (e.g. sepsis, pneumonia, subcutaneous infection urinary tract infection)
Hypertension	+	−	−	−	+ (at 6 months of age)	−
Malformations	−	Atrial septum defect	−	−	Ear tag	Atrial septum defect
Cardiomegaly	+	+	+	−	-	−
Renal enlargement	+	−	−	−	+	−
Prognosis	Died at 4 months of age (respiratory failure with sepsis)	Alive at 2 years of age	Alive at 17 years of age	Died at 12 years of age (fulminant parvovirus B19-associated myocarditis)	Died at 10 months of age (respiratory insufficiency related to a respiratory syncytial virus)	Died at 2 years of age (respiratory failure and presumed sepsis)
ADAM17 alterations	Compound heterozygous missense variants	Compound heterozygous missense variants	Homozygous frame-shift variant (4 bp deletion)	Homozygous frame-shift variant (4 bp deletion)	Homozygous frame-shift variant (1 bp insertion)	Homozygous gross deletion (exon 1)
Reference	−	−	^5^	^5^	^6^	^7^

*GA* gestational age.

To date, the reported pathogenic genetic deficiency of *ADAM17* in NISBD1 cases included a homozygous frameshifting 4 bp- or 1 bp-insertion variant producing a premature termination codon and a homozygous gross deletion including exon 1 (Table 1)^5–7^, resulting in the complete loss of ADAM17 expression through nonsense-mediated mRNA decay (NMD) and loss of transcription from exon 1, respectively. Although four additional null variants, which could cause NMD, have been reported in the ClinVar database (https://www.ncbi.nlm.nih.gov/clinvar; updated 2021, January 10), no missense pathogenic variants of *ADAM17* have been reported to cause NISBD1.

Recently, we identified compound heterogeneous missense *ADAM17* variants through targeted panel sequencing (TPS) in the Japanese male infant with erythroderma and exudate in whole body, recurrent skin infection, pneumonia and sepsis and prolonged diarrhoea. Same compound heterogeneous missense *ADAM17* variants were detected in his younger brother with similar clinical features. Because both missense variants were present outside the catalytic domain of ADAM17 and both mutants seemed to be equally transcribed, experimental evidence supporting loss of catalytic activity of the individual ADAM17 mutant was necessary to molecularly diagnose NISBD1^8^.

Here, we report for the first time, a family with pathogenic missense variants in *ADAM17* responsible for NISBD1 in a compound heterozygous state by providing experimental evidence of their damaging effects on the ectodomain shedding activity of ADAM17 using the in vitro functional assay system optimised for non-synonymous missense variants detected in *ADAM17*.

## Materials and methods

### TPS

TPS for genomic DNA extracted from peripheral blood lymphocytes was performed using the TruSight One Sequencing Panel (Illumina, San Diego, CA, USA) and a MiSeq sequencer (Illumina) according to the manufacturer’s instructions, followed by our pipeline for NGS data analysis as described^9^ with a minor modification due to a software update specific for the bioinformatics pipeline^10^. To identify pathogenic single nucleotide variants (SNVs), we excluded sequence variants with low-allele frequencies (> 0.01) in the 1000 Genomes Project database (http://www.1000genomes.org), National Heart, Lung and Blood Institute Grand Opportunity (NHLBI GO) Exome Sequencing Project (ESP6500, http://evs.gs.washington.edu/EVS), The Genome Aggregation Database (gnomAD, https://gnomad.broadinstitute.org/), Human Genetic Variation Database (HGVD, http://www.genome.med.kyoto-u.ac.jp/SnpDB) and Japanese Multi Omics Reference Panel (jMorp, https://jmorp.megabank.tohoku.ac.jp/202008/). Copy-number variation (CNV) analysis using TPS data was performed as described^10,11^.

### Primers

Primers used in this study are listed in Supplementary Table S1.

### PCR, reverse transcription-PCR and quantitative RT-PCR

Genomic DNA was extracted from peripheral blood leucocytes using the Gentra Puregene Cell kit (Qiagen, Hilden, Germany). Total RNA was extracted from peripheral blood leucocytes and cultured cells using the RNAiso Plus kit (Takara Bio, Kusatsu, Japan) and reverse-transcribed using PrimeScript reagent kit (Takara Bio) for synthesising complementary DNA (cDNA).

PCR and reverse transcription-PCR (RT-PCR) were performed using genomic DNA and cDNA, respectively and PrimeSTAR^®^ GXL DNA Polymerase (Takara Bio), according to the manufacturer’s protocols.

For quantification of mRNA levels, quantitative RT-PCR (qRT-PCR) was performed as described using specific primer sets with the SYBR Green Master Mix (Applied Biosystems, Waltham, MA, USA) or TaqMan kit (Applied Biosystems)^12^. For normalisation, glyceraldehyde-3-phosphate dehydrogenase (*GAPDH*) mRNA was used as an internal control.

### Sanger sequencing

Direct Sanger sequencing of PCR products was performed using the BigDye Terminator v3.1 Cycle Sequencing Kit (Applied Biosystems) and ABI 3500xL Genetic Analyzer (Applied Biosystems).

### Cell lines

Wild-type (WT) and *Adam10* and *Adam17* double-knockout mouse embryonic fibroblasts (*Adam10*/*17*^−/−^ mEFs)^13^ from Horiuchi and human embryonic kidney 293 (HEK293) cells, were grown in Dulbecco’s modified Eagle’s medium (DMEM) supplemented with foetal bovine serum and antibiotics.

### ADAM17 expression plasmid

The codon-optimised synthetic DNA fragment encoding full-length human ADAM17 (Supplementary Figure S1; Thermo Fisher Scientific, Waltham, MA, USA) was inserted into the pCMV-3Tag-3A vector (Agilent Technologies; Santa Clara, CA, USA) between *Xho*I and *Bam*HI sites (pFLAG-ADAM17-WT) to append three tandem FLAG epitopes to the C-terminus of ADAM17. To obtain plasmids expressing mutant ADAM17 (pFLAG-ADAM17-C567R and pFLAG-ADAM17-C600Y), site-directed mutagenesis was performed using the KOD-Plus-Mutagenesis kit (Toyobo, Osaka, Japan) according to the manufacture’s protocol.

Expression plasmids of ADAM17 with the HiBiT sequence inserted between metalloprotease and disintegrin domains (pHiBiT-ADAM17-WT, pHiBiT-ADAM17-C567R and pHiBiT-ADAM17-C600Y, Supplementary Figure S2a) were constructed through inverse PCR with specific primers using pFLAG-ADAM17-WT, pFLAG-ADAM17-C567R and pFLAG-ADAM17-C600Y as templates, respectively.

### Transient transfection experiments

Expression plasmids and their control plasmids were separately transfected into HEK293 cells and *Adam10*/*17*^−/−^ mEFs using Lipofectamine 2000 (Invitrogen, Carlsbad, CA, USA) and Neon Transfection System (Invitrogen), respectively, according to the manufacturer’s protocol.

### Antibodies

The antibodies used in this study are listed in Supplementary Table S2.

### Western blot analysis

Cell lysates were prepared using the radioimmunoprecipitation (RIPA) buffer (Nacalai Tesque, Kyoto, Japan) or M-PER Mammalian Protein Extraction Reagent (Thermo Fisher Scientific) supplemented with the protease inhibitor cocktail (Nacalai Tesque). Western blot analysis was performed as described^12^. Images were obtained with the GE Amersham Imager 600 (GE Healthcare, Milwaukee, WI, USA) or FUSION SOLO.7S.EDGE (Vilber-Lourmat, Marne la Vallée, France).

### Cell-based shedding assay

Plasmids encoding alkaline phosphatase (AP)-tagged partial human TNF-α, TGF-α and HB-EGF expression constructs (AP-TNF-α, AP-TGF-α and AP-HB-EGF, respectively) from Higashiyama were co-transfected with pFLAG-ADAM17-WT, pFLAG-ADAM17-C567R, or pFLAG-ADAM17-C600Y into *Adam10*/*17*^*−/−*^ mEFs^14,15^. After 48 h, cells were cultured in serum-free DMEM medium for 1 h and then in serum-free DMEM with either 100 nM phorbol 12-myristate 13-acetate (PMA) or 4 μM batimastat (BB94) or both for 2 h. For the specific inhibition of proteolysis of ADAM17 substrates in culture cells, 15 μg/mL of human ADAM17 inhibitory antibody D1 (A12) instead of BB94 was also used in the similar protocol. Unstimulated cells (vehicle alone) were treated with solvent (dimethyl sulphoxide, DMSO or saline). AP activity was determined through colorimetry^16^.

### Protein decay assay

To measure the relative ADAM17 protein stabilities in HEK293T cells transfected with pFLAG-ADAM17-WT, pFLAG-ADAM17-C567R, or pFLAG-ADAM17-C600Y and incubated for 48 h, cells were exposed to the translational inhibitor cycloheximide (0.1 mg/mL) (Sigma-Aldrich, St Louis, MO, USA) for indicated times. Subsequently, FLAG-syn-hADAM17 levels were detected by western blotting using an anti-FLAG antibody and band intensities corresponding to ADAM17 were quantified. β-actin was used as a loading control. Values are expressed as fold changes compared with those measured at time 0.

### Maturation experiments

Regarding maturation experiments for ADAM17, *Adam10*/*17*^*−/−*^ mEFs were transfected with pFLAG-ADAM17-WT, pFLAG-ADAM17-C567R, or pFLAG-ADAM17-C600Y and incubated for 48 h. For PMA stimulation, cells were incubated with 100 nM PMA for 5 min and washed with PBS. After subsequent incubation at 37 °C in DMEM for indicated times, cells were harvested and lysed using RIPA buffer (Nacalai Tesque). Exogenous ADAM17 was detected using the anti-DDDDK antibody, as described^9^.

### Surface biotinylation assay

For the biotinylation of cell-surface proteins^17^, HEK293 cells were transfected with pFLAG-ADAM17-WT, pFLAG-ADAM17-C567R, or pFLAG-ADAM17-C600Y and incubated for 48 h. For PMA stimulation, cells were incubated with 100 nM PMA for 5 min and washed with PBS. After subsequent incubation at 37 °C in DMEM, cells were washed three times with ice-cold PBS (pH 8.0) and incubated with EZ-Link Sulfo-NHS-LC-Biotin (1.0 mg/mL) (Thermo Fisher Scientific) in PBS (pH 8.0) for 30 min at 25 °C. Cells were lysed with the RIPA buffer (Nacalai Tesque) on ice for 10 min. Then, biotinylated proteins (2 mg) were incubated with 40 μL streptavidin beads for 1 h at 4 °C, beads were washed three times with PBS (pH 7.4) and heated in 30 μL 2 × Laemmli buffer for western blot analysis.

### NanoLuc Binary Technology (NanoBiT) complementation assay

For quantifying HiBiT-tagged ADAM17 expression on the cell surface, the Nano-Glo HiBiT Extracellular Detection System (Promega, Madison, WI, USA) and Nano-Glo HiBiT Lytic Detection System (Promega) were used according to the manufacturer’s instructions^18^. *Adam10*/*17*^*−/−*^ mEFs transfected with pHiBiT-ADAM17-WT, pHiBiT-ADAM17-C567R, or pHiBiT-ADAM17-C600Y for 48 h in 96-well microplates were incubated with PMA for 5 min and washed with PBS. After subsequent incubation at 37 °C in DMEM for 2 h, the cell culture medium in each well was replaced by 100 μL of Nano-Glo HiBiT Extracellular Reagent, and the plate was incubated at 25 °C for 10 min (Supplementary Figure S2b). Luciferase activity was measured using a SpectraMax i3x microplate reader (Molecular Devices). Then, cells were washed with PBS three times and 100 μL of Nano-Glo HiBiT Lytic Reagent was added to the well. After incubation at 25 °C for 10 min, luciferase activity was measured in each sample. The ratio of extracellular and lytic luminescence intensity for each well was calculated.

### Statistical analysis

Differences among subgroups were tested with Student’s *t* test or with analysis of variance and Tukey’s multiple comparison test. Differences were assessed with a two-sided test and considered significant at *P* < 0.05.

### Ethical approval

We obtained written informed consent from both parents of patients for study participation and publication of identifying information in an online open-access publication. The study was performed according to protocols of the Declaration of Helsinki and was approved by the ethics committee of Tokushima University.

## Results

### Case presentation

Two brothers, born to non-consanguineous healthy Japanese parents, presented similar clinical manifestations, including no hair, thin eyebrows, erythroderma with exudate in whole body, intractable diarrhoea, failure to thrive, mild cardiomegaly, lymphadenopathy, eosinophilia, hyper-immunoglobulin E and recurrent sepsis from early postnatal period (Table 1). Their mother conceived through in vitro fertilisation both times because of bilateral oviductal obstruction and ovarian dysfunction. The elder brother (proband) was born at 32 weeks of gestational age by caesarean section due to growth arrest and breech presentation with a reduced birth weight of 1144 g (0.3 percentile). He died at 4 months of age due to respiratory failure associated with pneumonia and sepsis. The younger brother was born at 37 weeks of gestational age by repeat caesarean section with a reduced birth weight of 2066 g (0.9 percentile) and is alive at 2 years of age with a reduced weight of 7 kg (− 3.5SD). He exhibited severe failure to thrive requiring tube feeding with elemental diet and repeated infections, including sepsis, pneumonia and skin infection, treated with intravenous immunoglobulins, oral steroids and antimicrobial prophylaxis. Analysis of skin biopsy samples from the elder brother revealed parakeratosis, infiltration of neutrophils and lymphocytes and strong spongy oedema in the stratum corneum of the epidermis and lymphocyte and neutrophil infiltration in the dermis. The elder brother alone exhibited high-renin hypertension with bilateral enlargement of kidneys. The younger brother required administration of diuretics due to atrial septum defect with pulmonary hypertension. Details of the clinical history of the elder brother (proband) are shown in Supplementary data.

### Identification of *ADAM17* compound heterozygous missense variants in affected infants

**Figure 1:**
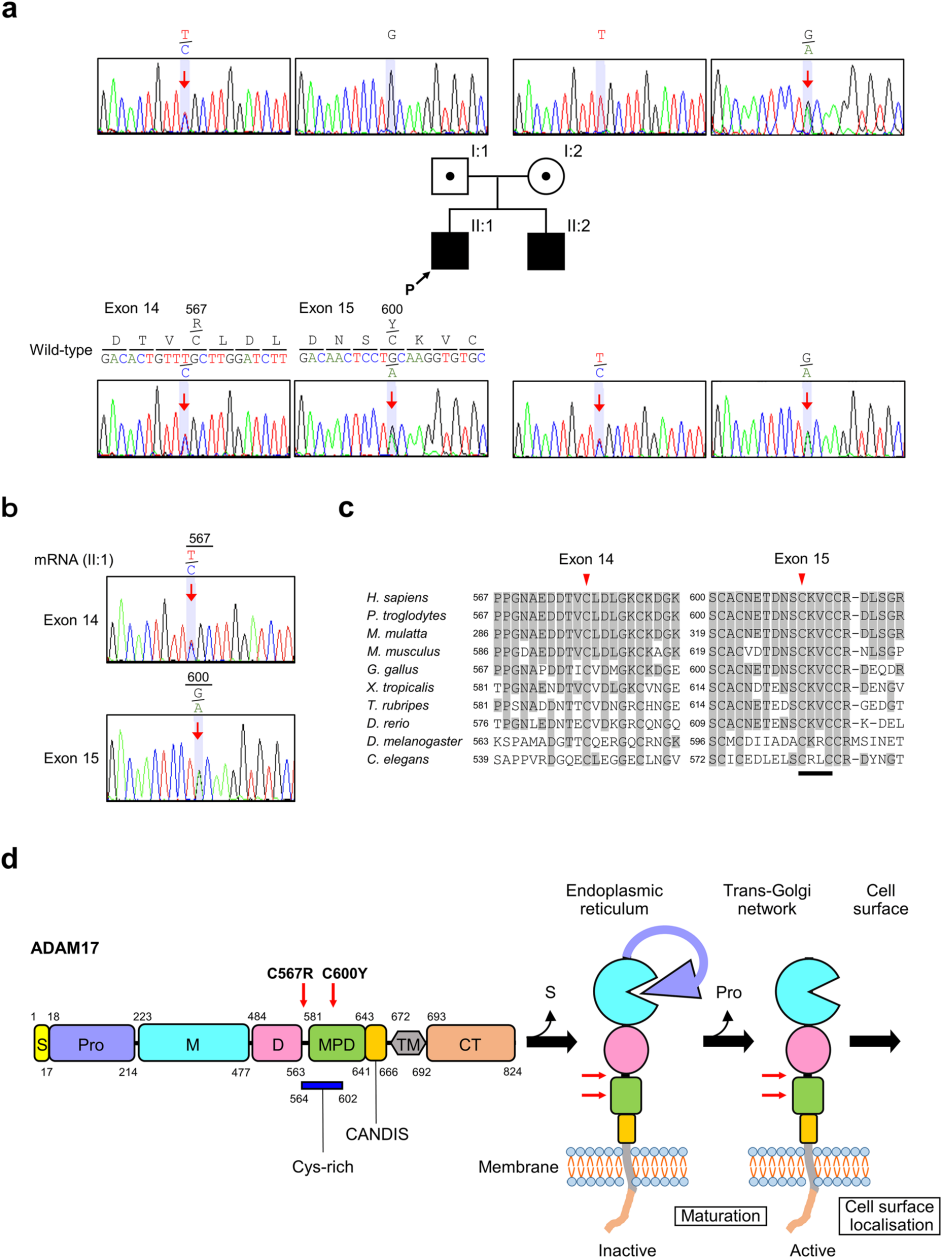
(**a**) Partial sequence chromatograms of exons 14 and 15 of a disintegrin and metalloprotease 17 (*ADAM17*) in the genomic DNA of patients (II:1 and II:2) and their parents (I:1 and I:2). Nucleotide and corresponding amino acid sequences of WT and mutant ADAM17 are also shown. Numbers indicate transformed codons by single-base substitutions (red arrows). *P* proband. (**b**) Partial sequence chromatograms of exons 14 and 15 of *ADAM17* in the mRNA of peripheral blood leucocytes from the proband (patient II:1). Both WT and variant alleles were detected at the same level, suggesting that *ADAM17* was equally transcribed from both alleles. Numbers indicate transformed codons by single-base substitutions (red arrows). (**c**) Comparison of partial amino acid sequences of ADAM17 in human (NP_003174.3), chimp (XP_515293.2), rhesus monkey (XP_002799185.1), mouse (NP_033745.4), chicken (NP_001008682.1), *Xenopus tropicalis* (NP_001182159.1), fugu (XP_011616093.2), zebrafish (Adam17a, NP_955967.1), fruit fly (Tace, NP_733334.1) and *Caenorhabditis elegans* (adm-4, NP_509318.1). Conserved amino acids are highlighted. Mutated amino acids are shown by red arrowheads. Numbers indicate codons of mutated amino acids. Bar indicates the highly conserved thioredoxin CXXC motif. (**d**) Schematic representation of ADAM17. Domains are depicted approximately to the scale adapted from the human reference amino acid sequence (NP_003174.3). Red arrows show the position corresponding to variants detected in patients in a compound heterozygous state and both parents in a heterozygous state. *CANDIS* conserved ADAM17 dynamic interaction sequence, *MPD* membrane proximal domain, *CT* C-terminal cytoplasmic tail, *D* disintegrin-like domain, *M* metalloprotease domain, *Pro* pro-domain, *S* signal sequence, *TM* transmembrane domain. Blue bar indicates the cysteine-rich (Cys-rich) region. The ADAM17 precursor (left) is converted into a mature active form (right) through the enzymatic removal of the signal sequence and pro-domain. Activated ADAM17 is trafficked to the cell surface, where shedding of substrates can occur.

To search for the genetic cause, we performed TPS of exon regions of 4813 clinically relevant genes, using genomic DNA extracted from the proband’s blood sample, to detect SNVs, insertion-deletions (InDels) and CNVs simultaneously and prioritisation of genetic variants^9–11^. Although no possible disease-causing variants were detected within coding regions of candidate genes responsible for severe combined immunodeficiency, such as *DCLRE1C*, *IL2RG*, *RAG1*, *RAG2* and *RMRP*, in the TPS panel, two single-base substitutions, namely NM_003183.6:c.1699T>C (p.Cys567Arg or p.C567R) and c.1799G>A (p.Cys600Tyr or p.C600Y), in exons 14 and 15 of *ADAM17*, respectively, were detected. Both substitutions were confirmed by Sanger sequencing (Fig. 1a). The proband’s father and mother were heterozygous carriers of c.1699T>C and c.1799G>A, respectively, suggesting that these variations were present in the proband in a compound heterozygous state (Fig. 1a). Both alleles were almost equally transcribed endogenously in peripheral blood cells (Fig. 1b). No cases with these variants are present in population databases, such as 1000 Genomes, ESP6500, gnomAD, HGVD and jMorp 8.3KJPN. Moreover, these variants are not listed in Human Gene Mutation Database (HGMD, Professional 2020.3; http://www.hgmd.org/) or ClinVar. Multiple sequence alignments of ADAM17 orthologs across species revealed that both missense variants affect highly conserved residues (Fig. 1c). Both Cys567 and Cys600 are located outside the functional catalytic domain, but within the compositional bias region annotated ‘Cys-rich’ (UniProt, https://www.uniprot.org/, Fig. 1d). Bioinformatics tools, including MutationTaster2 (http://www.mutationtaster.org/index.html), PolyPhen-2 (version 2.2.2r405b, http://genetics.bwh.harvard.edu/pph2/index.shtml), PROVEAN (version 1.1, http://provean.jcvi.org/seq_submit.php), SIFT (version 1.03, http://sift.jcvi.org/) and PANTHER (version 1.02, http://www.pantherdb.org/tools/csnpScoreForm.jsp), predicted p.Cys567Arg as disease-causing (0.999), probably damaging (1.0), deleterious (− 10.404), deleterious (0.0) and deleterious (− 6.89567), respectively. By contrast, p.Cys600Tyr was predicted to be disease-causing (0.999), deleterious (− 10.228), deleterious (0.0) and deleterious (− 5.04949) by MutationTaster PROVEAN, SIFT and PANTHER, respectively, and benign (0.419) by Polyphen-2. Cys600 locates within the membrane proximal domain (MPD), whereas Cys567 locates between the metalloproteinase domain and the MPD (Fig. 1d). Although no pathogenic missense variants in human *ADAM17* were reported in patients with NISBD1, p.Cys600Tyr was detected in the subline without sheddase activity established from mutagenised Chinese hamster ovary (CHO) cells^19^. In addition, the functional importance of Cys600 for the shedding activity of ADAM17, with possible mechanisms, has been demonstrated using full-length murine or partial human recombinant proteins in vitro^20–22^.

TPS analysis and re-evaluation of clinical features of the affected proband and his unaffected parents revealed that the proband may have NISBD1 caused by novel pathogenic missense variants of *ADAM17* in a compound heterozygous state. Same variants of *ADAM17* in a compound heterozygous state were detected in the second child having similar clinical features. However, alterations of human *ADAM17* observed in all reported NISBD1 cases seem to cause a complete loss of ADAM17 expression by inducing NMD or loss of transcription from exon 1^5–7^. According to the American College of Medical Genetics and Genomics (ACMG)/Association for Molecular Pathology clinical variant interpretation guidelines^8^, NM_003183.6:c.1699T>C and c.1799G>A are classified ‘Uncertain significance’ (PM2, PM3, PP3 and BP1) and ‘likely pathogenic’ (PS3, PM2 and BP1), respectively. Therefore, to confirm the pathogenicity of these missense variants, especially p.Cys567Arg, in vitro or in vivo functional evidence supporting a damaging effect on the gene product (PS3) are necessary^8,23^. Although in vivo cytokine production experiments using peripheral blood mononuclear cells (PBMCs) from patients and age-matched controls may be useful to assess the function of the endogenous mutant ADAM17 proteins^5^, it is difficult to obtain sufficient amounts of PBMCs from infants for in vivo cytokine production experiments in our cases. Therefore, we tested whether these missense variants significantly affect the enzymatic activity of ADAM17 and are responsible for clinical features of our patients using cells exogenously introduced ADAM17 variants.

### p.Cys567Arg and p.Cys600Tyr variants affect the enzymatic activity of ADAM17

To study the effects of each variant on the catalytic activity of ADAM17, we exogenously introduced epitope-tagged WT or mutants of human ADAM17 into *Adam10*/*17*^−/−^ mEFs^13^. ADAM10 can constitutively shed various substrates of ADAM17 under chronically ADAM17-deficient conditions^13^. Therefore, we used *Adam10*/*17*^−/−^ mEFs instead of *Adam17*^−/−^ mEFs to prevent any background sheddase activity for substrates of ADAM17, although ADAM17 is considered to be the primary sheddase for its substrates, especially when stimulated with PMA^13^. Although the full-length coding sequence of human *ADAM17* could be amplified from RNA extracted from human cells through RT-PCR, it was impossible to clone PCR products into plasmids and stably replicate them in *Escherichia coli* due to the spontaneously and frequently occurring SNVs, InDels and/or rearrangements in *ADAM17*, as warned by OriGene Technologies (Rockville, MD, USA; https://www.origene.com/catalog/cdna-clones/expression-plasmids/sc316426/adam17-nm_003183-human-untagged-clone). Therefore, we synthesised an artificial DNA fragment (syn-hADAM17-WT) containing the optimised nucleotide sequence, which is different from the reference sequence (NM_003183.5) but encodes the same amino acid sequence as WT human ADAM17 (hADAM17-WT, NP_003174.3, Supplementary Figure S1).

**Figure 2:**
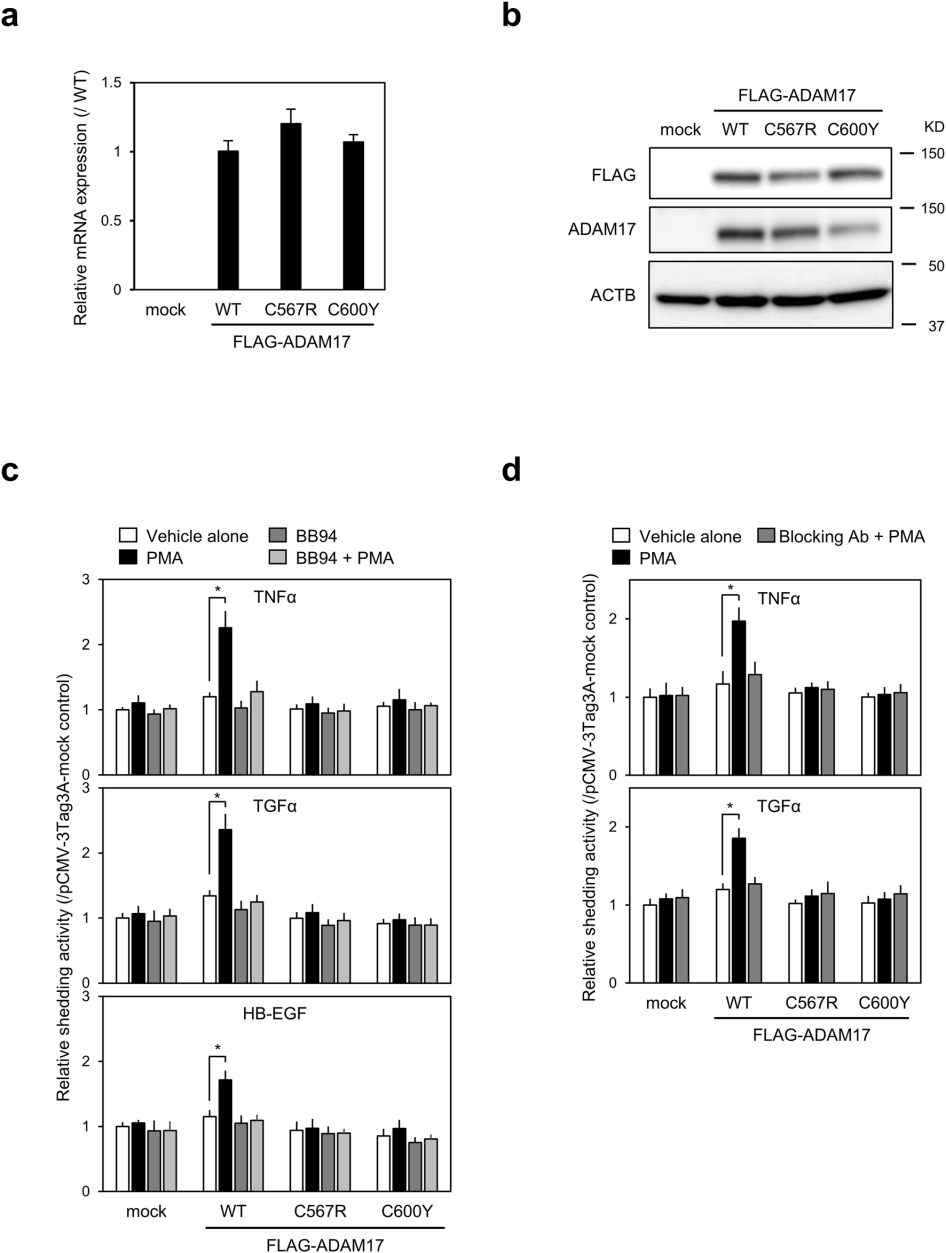
(**a**,**b**) mRNA and protein expression of exogenously transfected a disintegrin and metalloprotease 17 (*ADAM17*) in *Adam10/17* double-knockout mouse embryonic fibroblasts (*Adam10*/*17*^−/−^ mEFs). (**a**) *Adam10/17*^−/−^ mEFs were transfected with FLAG-syn-hADAM17-WT, FLAG-syn-hADAM17-C567R, or FLAG-syn-hADAM17-C600Y and incubated for 48 h. Amounts of exogenous *ADAM17* mRNA were measured by qRT-PCR, using *GAPDH* mRNA as an endogenous control. Values are expressed as fold changes (mean ± SD, n = 3) compared with respective values in cells transfected with syn-hADAM17-WT. (**b**) Transfected cells were lysed in M-PER buffer supplemented with a protease inhibitor. Panels show representative results from three independent western blots analysing the expression of FLAG-tagged ADAM17 and β-actin. Full-length blots were presented in Supplementary Figure 5a (**c**,**d**) Phorbol 12-myristate 13-acetate (PMA)-stimulated shedding activity for various substrates in *Adam10/17*^*−*/−^ mEFs exogenously transfected with ADAM17. (**c**) *Adam10/17*^−/−^ mEFs were co-transfected with pFLAG-syn-hADAM17 expression constructs and AP-tagged TNF-α, TGF-α, or HB-EGF expression constructs. Relative AP activity released into the media of transfected *Adam10/17*^−/−^ mEFs during the 2-h stimulation with PMA (20 ng/mL) in the presence or absence of the metalloprotease inhibitor BB94 (1 μM). Data represent three independent experiments. Error bars indicate mean ± SD. *P < 0.05. (**d**) *Adam10/17*^−/−^ mEFs were co-transfected with pFLAG-syn-hADAM17 expression constructs and AP-tagged TNF-α or TGF-α expression constructs. Relative AP activity released into the media of transfected *Adam10/17*^−/−^mEFs during the 2-h stimulation with PMA (20 ng/mL) in the presence or absence of the human ADAM17 inhibitory antibody (15 μg/mL). Data represent three independent experiments. Error bars indicate mean ± SD. *P < 0.05.

Expression constructs containing syn-hADAM17-WT (FLAG-syn-hADAM17-WT), syn-hADAM17 encoding mutant ADAM17 harbouring the p.Cys567Arg variant (FLAG-syn-hADAM17-C567R) and syn-hADAM17 encoding mutant ADAM17 harbouring the p.Cys600Tyr variant (FLAG-syn-hADAM17-C600Y) with three tandem FLAG epitopes at the C-terminus were generated and transfected into *Adam10/17*^−/−^ mEFs. No difference in viability was observed among cells transiently transfected with each construct, and almost equal amounts of mRNA expression were detected from transfected ADAM17 constructs (Fig. 2a). Moreover, ADAM17 was almost equally expressed from all three constructs at the expected size (Fig. 2b).

Because ADAM17 is responsible for the proteolytic cleavage of membrane-anchored precursor substrates in a process defined as ectodomain shedding, we determined the effects of each variant on ADAM17 sheddase activity.

We established a sensitive cell-based method to determine ADAM17 sheddase activity. We co-transfected AP-TNF-α, AP-TGF-α, or AP-HB-EGF expression constructs with pFLAG-syn-hADAM17-WT, pFLAG-syn-hADAM17-C567R, or pFLAG-syn-hADAM17-C600Y into sheddase activity-free *Adam10/17*^−/−^ mEFs. PMA-stimulated soluble AP-substrate release was detected in cells expressing FLAG-syn-hADAM17-WT, but not in cells expressing either FLAG-syn-hADAM17-C567R or FLAG-syn-hADAM17-C600Y for AP-tagged substrate (Fig. 2c). This PMA-induced soluble AP-substrate release by co-transfection with FLAG-syn-hADAM17-WT was inhibited by a 2 h-pretreatment with the metalloprotease inhibitor BB94 (Fig. 2c). No significant effects on PMA-induced soluble AP-substrate release were observed in FLAG-syn-hADAM17-C567R- or FLAG-syn-hADAM17-C600Y-co-transfected cells through BB94 treatment^16^. The same findings were obtained upon treatment with a human ADAM17 inhibitory antibody D1(A12), which binds to both catalytic and noncatalytic domains of ADAM17 and specifically inhibits the proteolysis of ADAM17 substrates in cell culture (Fig. 2d)^24^. Our results suggest that the two mutant proteins, namely hADAM17-C567R and hADAM17-C600Y, lack PMA-stimulated cell-based ectodomain shedding activity for TNF-α, TGF-α and HB-EGF, supporting the pathogenicity of the two variants detected here by providing in vitro functional evidence of their damaging effects on the gene products (PS3)^8^.

### p.Cys567Arg and p.Cys600Tyr variants do not affect cell-surface localisation of ADAM17 after stimulation

Although both ADAM17 mutants showed reduced PMA-stimulated shedding activity in a cell-based assay, both variants do not locate within the catalytic domain. As shown in Fig. 2a,b, mRNA and protein expression levels of both ADAM17 mutants were similar to those of WT ADAM17, respectively. In addition, no difference in stability of exogenously expressed ADAM17 was observed among *Adam10/17*^−/−^ mEFs transfected with each ADAM17 expression construct 12 h after cycloheximide treatment (Supplementary Figure S3). These results suggest that both variants do not affect the transcription, translation and stability of ADAM17.

**Figure 3:**
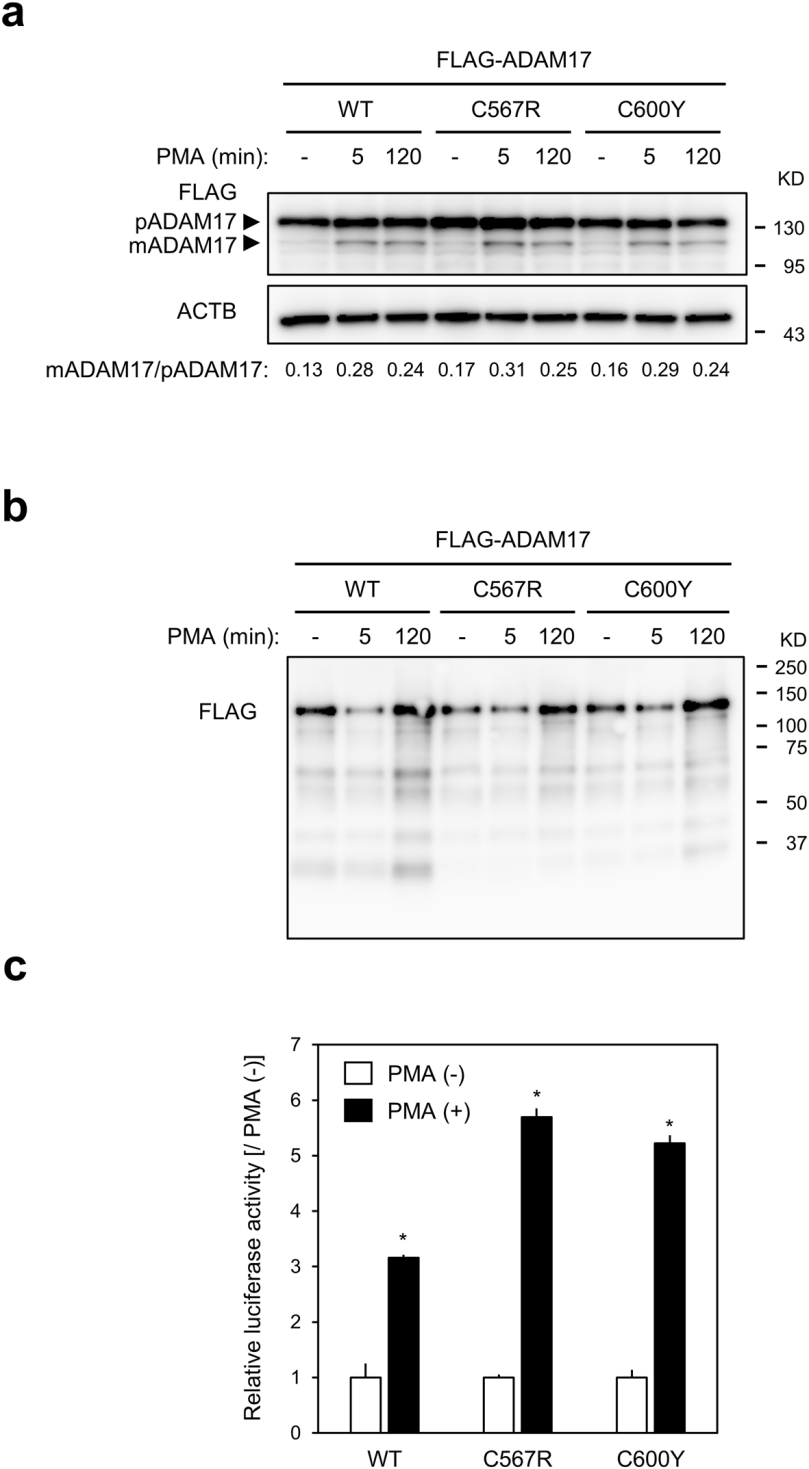
(**a**) Effect of variants on the phorbol 12-myristate 13-acetate (PMA)-induced maturation of a disintegrin and metalloprotease 17 (ADAM17). *Adam10/17* double-knockout mouse embryonic fibroblasts (*Adam10/17*^*−/−*^ mEFs) were transfected with pFLAG-syn-hADAM17 expression constructs, incubated for 48 h and treated with PMA (100 ng/mL) in serum-free medium for 5 min. After subsequent incubation at 37 °C in DMEM for indicated times, cells were lysed in RIPA buffer supplemented with a protease inhibitor. Panels show representative results from three independent western blots analysing the expression of FLAG-tagged mature ADAM17 (mADAM17) and its preform (pADAM17) and an internal control β-actin (ACTB). The ratio of the densitometric signal of mADAM17 and pADAM17 was shown at the botttom. Full-length blots were presented in Supplementary Figure S5b. (**b**) Effect of variants on PMA-induced cell-surface localisation of mature ADAM17. HEK293 cells transfected with pFLAG-syn-hADAM17 expression constructs and incubated for 48 h were treated with PMA (100 ng/mL) in serum-free medium for 5 min. After incubation at 37 °C in DMEM for indicated times, cell-surface proteins were biotinylated and harvested. Biotinylated proteins precipitated using streptavidin beads were immunoblotted. The panel shows the representative result from three independent experiments. A full-length blot with a size marker image was presented in Supplementary Figure S5c. (**c**) Quantification of HiBiT-tagged ADAM17 localised on the cell surface. *Adam10*/*17*^*−/−*^ mEFs transfected with pHiBiT-ADAM17-WT, pHiBiT-ADAM17-C567R, or pHiBiT-ADAM17-C600Y and incubated for 48 h in 96-well microplates were treated with PMA (20 ng/mL) for 2 h. Extracellular and lytic luciferase activities were measured as described in the “Materials and methods” section (Supplementary Figure S2B). Values are expressed as the ratio of extracellular or lytic luminescence intensity for each well (mean ± SD, n = 6). *P < 0.05. Data are representative of at least three experiments with similar results.

Another possible mechanism contributing to the catalytic activity of ADAM17 is its cellular localisation after stimulation (Fig. 1d)^17^. To test this directly, we evaluated the cell-surface abundance of mature ADAM17. We first analysed the maturation of exogenously transfected ADAM17 in HEK293 cells at different time points after PMA stimulation (Fig. 3a). An increase in mature ADAM17 at 5 min followed by a slight decrease at 120 min after PMA treatment was observed in FLAG-syn-hADAM17-WT-transfected cells. A similar change was observed in FLAG-syn-hADAM17-C567R- and FLAG-syn-hADAM17-C600Y-transfected cells. We next analysed the cell-surface expression of exogenously transfected ADAM17 using biotinylation of surface proteins at different time points after PMA stimulation (Fig. 3b). At 120 min after PMA treatment, a drastic increase in biotinylated cell surface ADAM17 was observed in FLAG-syn-hADAM17-WT-, FLAG-syn-hADAM17-C567R- and FLAG-syn-hADAM17-C600Y-transfected cells. This finding was confirmed using the NanoBiT complementation assay^18^. Expression plasmids encoding HiBiT-tagged ADAM17-WT, hADAM17-C567R and hADAM17-C600Y, in which HiBiT was inserted in the extracellular region between the metalloprotease and disintegrin domains of ADAM17, were transfected into *Adam10/17*^−/−^ mEFs (Supplementary Figure S2a). In the presence of the large NanoLuc subunit (LgBiT), complementation occurs between HiBiT and LgBiT, reconstituting a full-length functional NanoLuc luciferase (Supplementary Figure S2b)^18^. Cell-surface localisation of exogenously expressed HiBiT-tagged ADAM17-WT, hADAM17-C567R and hADAM17-C600Y was detected as an increase in luminescence by adding LgBiT with the substrate furimazine extracellularly relative to that by adding LgBiT with the substrate furimazine into total cell lysate (Fig. 3c). Thus, the lack of PMA-stimulated ectodomain shedding activity in the two mutant proteins, hADAM17-C567R and hADAM17-C600Y, is unlikely to be caused by impaired PMA-induced maturation and trafficking to the cell-surface of ADAM17 (Fig. 1d).

## Discussion

We identified siblings, with erythroderma and exudate in whole body, recurrent skin infection and sepsis and prolonged diarrhoea from the early neonatal period, harbouring compound heterozygous missense variants of *ADAM17*. Although both affected siblings manifested clinical features of NISBD1, all reported genetic deficiencies of *ADAM17* detected in patients with NISBD1^5–7^ and the ClinVar database included alterations causing a complete loss of ADAM17 expression and function (predicted null variants). No missense variants in the ClinVar database were classified as pathogenic or likely pathogenic, and most are referred to as ‘variants of uncertain/unclassified significance’ (VUS)^25^. NGS generates a huge array of genetic changes and an increasing number of unsolved cases carrying VUS with limited pathogenicity predictions exist due to limitations of in silico prediction tools and our current state of knowledge. In the ClinVar database (updated 2021, January 10), 66 ADAM17 missense variants detected in patients with NISBD1 as a clinical condition were reported; all were interpreted as benign (two variants), likely benign (one variant), or VUS (66 variants). Kosukcu et al*.* reported results of whole-exome sequencing in 11 unclassified autoinflammatory diseases and identified one homozygous missense variant in ADAM17, namely NM_003183.4:c.851T>C; p.Ile284Thr, in patients with severe pustular skin lesions and nail abnormalities similar to NISBD1^26^. However, this missense variant was also unable to be classified as pathogenic or likely pathogenic due to lack of functional evidence (PM2, PP1 and PP3). Thus, there is a need for validation studies using established functional assays for molecular diagnosis of NISBD1. Because *ADAM17* encodes a major sheddase with catalytic activity, we set up in vitro enzymatic assay, namely cell-based shedding assay, for WT and mutant ADAM17 to interpret the functional consequences of *ADAM17* missense variants identified in our patients. In this study, we experimentally confirmed the lack of ectoderm sheddase activity of exogenously expressed ADAM17 with each missense variant observed in patients and molecularly diagnosed that patients were affected by NISBD1 caused by novel pathogenic missense variants in* ADAM17*. Our findings suggest that this rare syndrome can be caused by various types of alterations, including missense variants, in both alleles of *ADAM17* and cause loss-of-function of ADAM17. In addition, our functional assay system may be useful to evaluate the pathogenicity of *ADAM17* missense variants and contribute to the accurate diagnosis of NISBD1.

ADAM17 mediated shedding was reported to be negatively regulated by extracellular protein disulfide isomerases^13,15^. Among two missense variants identified in the presented case, p.Cys600Tyr occurred in the highly conserved thioredoxin cysteine-X-X-cysteine (CXXC) motif within the MPD of ADAM17 (Fig. 1c,d), which is crucial for enzymatic activity and is a target for thiol isomerisation^19–22^. Cys600 also locates within the cysteine-rich region (codons 564–602), which includes a part of the disintegrin domain and MPD (Fig. 1d). Cys600 was reported to be crucial for the activity of full-length ADAM17 using the subline without sheddase activity established from mutagenised CHO cells^19^. Moreover, Cys600 contributes to structural changes of MPD, between active open and inactive closed conformations, as a molecular switch, facilitating the global reorientation of the extracellular domains of ADAM17 and regulating its shedding activity^19,20^. Cys600 binds to Cys635 in the active open conformation but binds to Cys630 in the inactive closed conformation^22^. In addition, the extracellular juxtamembrane region including MPD and the cysteine-rich region was described to be involved in substrate recognition through various models, such as disulfide bond exchange, interaction with charged phospholipids or interaction of inactive Rhomboids 1 and 2^22,27,28^. Therefore, it is possible that the cysteine to tyrosine change at codon 600 induces a conformational change or an altered interaction with regulatory partners in the extracellular juxtamembrane region, especially within the MPD, resulting in altered regulation of stimulation and/or substrate selectivity of ADAM17.

Functional evidence demonstrating the effect of Cys567 on the shedding activity of ADAM17 has not been reported. Cys567 also locates within the cysteine-rich region (codons 564–602) but outside the disintegrin domain and MPD (Fig. 1d). Therefore, Cys567Arg was classified as a VUS^8^, although p.Cys567Arg was predicted to be deleterious using multiple in silico predictive tools. Our cell-based shedding assay demonstrated that mutant ADAM17 having p.Cys567Arg lacks catalytic activity. Protein disulfide isomerase, which catalyses the formation and breakage of disulfide bonds between cysteine residues during protein folding, was demonstrated to potentially regulate cellular ADAM17 activity^29^. The MPD forms a functional unit that seems to be essential for regulating shedding by mediating substrate recognition and membrane binding^19–22,24,29–34^. The disintegrin domain, with the following N-terminus of the cysteine-rich region (C wrist, C_w_)^31^ before MPD, (Fig. 1d, Supplemental Figure S4a,b) was supposed to act as a scaffold and be responsible for a C-shape like structure of the extracellular domains of ADAM17 by bridging the catalytic domain and MPD, thereby ensuring rigidity of the extracellular region^30,31^. Therefore, it is possible that Cys567 contributes to the structure necessary for the shedding activity of ADAM17 by forming a disulfide bond. Consistently, a disulfide bond between Cys567 and Cys578 within the C_w_ region^31^ was predicted based on evidence from the structurally similar bovine ADAM10^30,31^ and the metalloproteinase VAP2B, which possesses a metalloproteinase/disintegrin/cysteine-rich domain, present in snake (*Crotalus atrox*) venom^35^. In addition, DiAminoacid Neural Network Application (DiANNA), a tool that predicts cysteine states of a protein forming a disulfide bond (http://bioinformatics.bc.edu/clotelab/DiANNA/)^36^, predicted disulfide bonds between Cys567 and Cys582 around the MPD of ADAM17. Although these findings suggest that Cys567 plays an important role in activating ADAM17 through disulfide bond formation, determining which cysteine residues bind Cys567 for activating ADAM17 is warranted.

Intramolecular disulfide bonds, a common feature of secretory proteins, are crucial for their structure, stability and function^37^. In the presented cases, both variants were observed in cysteine residues, which may affect the three-dimensional structure by preventing the formation of the highly conserved disulfide bonds important for maintaining the proper folding of ADAM17. We demonstrated that expression levels of ADAM17 mRNA and protein, degradation status of ADAM17, amount of matured ADAM17 after PMA treatment and cell-surface localisation status of active ADAM17 after PMA treatment were similar among WT and mutant ADAM17. Therefore, PMA stimulated-shedding activity of mutant ADAM17 may not be caused by the decreased expression of the matured or activated protein on the cell surface, but by the loss of catalytic activity of ADAM17 through the three-dimensional structural change-induced direct modification of the catalytic domain and/or inhibition of its interaction with substrates. In this study, we have provided insights into the genetic pathology of *ADAM17* in NISBD1 and established a functional assay system for its missense variants, which will be useful for the molecular diagnosis of this disease.

## Supplementary Information

Supplementary Information 1.
