# Supplementary material for: Functionally confirmed compound heterozygous ADAM17 missense loss-of-function variants cause neonatal inflammatory skin and bowel disease 1
Source: Sci Rep. 2021 May 5;11:9552. doi: 10.1038/s41598-021-89063-0 (PMC8100128; doi:10.1038/s41598-021-89063-0)
Supplement: Supplementary file 1 — Supplementary Information 1. [file 41598_2021_89063_MOESM1_ESM.docx]

**Supplementary information**

**Functionally confirmed compound heterozygous ADAM17 missense loss-of-function variants cause neonatal inflammatory skin and bowel disease 1**

Issei Imoto*, Masako Saito, Kenichi Suga, Tomohiro Kohmoto, Masanobu Otsu, Keisuke Horiuchi, Hironao Nakayama, Shigeki Higashiyama, Mayumi Sugimoto, Ayumi Sasaki, Yukako Homma, Miki Shono, Ryuji Nakagawa, Yasunobu Hayabuchi, Shoichiro Tange, Shoji Kagami, Kiyoshi Masuda

Issei Imoto*, iimoto@aichi-cc.jp, Aichi Cancer Center Hospital.

Masako Saito, m.saito@tokushima-u.ac.jp, Tokushima University

Kenichi Suga, suga.kenichi.1@tokushima-u.ac.jp, Tokushima University.

Tomohiro Kohmoto, tomo069medicalgenetic@gmail.com, Tokushima University.

Masanobu Otsu, otsumasanobu@gmail.com, Tokushima University.

Keisuke Horiuchi, keisukehoriuchi@gmail.com, Keio University.

Hironao Nakayama, hironao@hirokoku-u.ac.jp, Ehime University/

Shigeki Higashiyama, shigeki@m.ehime-u.ac.jp, Ehime University.

Mayumi Sugimoto, mayusugiped@gmail.com, Tokushima University.

Ayumi Sasaki, tommy84882000@yahoo.co.jp, Tokushima University.

Yukako Homma, farfalle31@hotmail.co.jp, Tokushima University.

Miki Shono, miki_m_m_5897@yahoo.co.jp, Tokushima University.

Ryuji Nakagawa, ryujina@tokushima-u.ac.jp, Tokushima University.

Yasunobu Hayabuchi, hayabuchi@tokushima-u.ac.jp, Tokushima University.

Shoichiro Tange, stange@sapmed.ac.jp, Tokushima University.

Shoji Kagami, kagami@tokushima-u.ac.jp, Tokushima University.

Kiyoshi Masuda, kiyoshim@med.kawasaki-m.ac.jp, Kawasaki Medical School

*Correspondence to: Issei Imoto, M.D., Ph.D.

**Supplementary Data**

**Detailed clinical history of the proband**

A Japanese male infant was born to non-consanguineous healthy parents at 32 weeks and 5 days of gestational age by urgent cesarean section due to growth arrest and breech presentation (Figure 1A). His mother was a 29-year-old primipara, who conceived after single embryo transfer following *in vitro* fertilisation because of ovarian dysfunction and bilateral oviductal obstruction. The Apgar scores were 9 and 10 at 1 and 5 minutes, respectively. He was admitted to the neonatal intensive care unit at Tokushima University Hospital for preterm birth. His birth weight was 1,148 g (0.3 percentile), length 36.0 cm (0.4 percentile), head circumference 28.8 cm (29.4 percentile). His skin was thin and brittle, giving the impression of immaturity for his gestational age. He had no hair and thin eyebrows. Dysmorphic features were not evident.

He presented with erythroderma and exudate in whole body after postnatal day 2. Because staphylococcal scalded skin syndrome was initially suspected, antibiotics were initiated. Chest X-ray showed mild cardiomegaly and cardiac ultrasonography showed transient thinning of the left ventricle wall. Abdominal ultrasonography revealed bilateral renal enlargement. Extensive itchy, scaling, and exfoliative erythroderma became prominent after postnatal day 15, and hypernatremia (Na^2+^ >160mEq/l) was exhibited due to insensible perspiration from the skin. Thick petrolatum was applied to reduce insensible perspiration and a large volume of infusion was required. He had frequent recurrent episodes of pneumonia and sepsis caused by staphylococcal species after postnatal day 28. Various antibiotics and antifungals were administered in addition to administration of granulocyte colony-stimulating factor and treatment for disseminated intravascular coagulation. Intravenous nutrition and elemental diet were required because of prolonged diarrhoea and poor weight gain. Hypertension (blood pressure 104/72 mmHg) with an increased plasma renin activity (>45 ng/ml/h) was noted. Urinary N-acetyl-β-D-glucosaminidase and urinary β2 microglobulin were elevated to 182.5 U/l (0.3-11.5) and 21,935 mg/ml (< 0.29), respectively. Axillary lymph nodes were enlarged and blood examinations showed eosinophilia (eosinophil 1,500-4,300 /μl) and a high level of immunoglobulin E (IgE; 1,156 IU/ml). Omenn syndrome was suspected because he presented with severe susceptibility to infection, erythroderma, failure to thrive, protracted diarrhea and lymphadenopathy. IgG, IgM and IgA levels were 944mg/dl, 67mg/dl and 59 mg/dl, respectively. Peripheral blood lymphocyte subsets were CD3 80.4% (76.2 ± 9.8), CD19 10.7% (11.7 ± 4.8), CD4 52.1% (52.0 ± 4.9), CD8 32.5% (20.4 ± 5.9), CD4/CD8 1.60 (1.46 ± 8.6), CD16 7.6% (5.9 ± 3.2), CD57 0.9% and HLA-DR 21.7% (28.3 ± 10.0). T-cell receptor excision circle (TREC) and Kappa-deleting recombination excision circle (KREC) were 7.8×10^3^ copies/μg DNA (5.8 ± 2.3×10^3^) and 7.8×10^3^ copies/μg DNA (7.9 ± 3.9×10^3^), respectively, at postnatal day 89. Histological analysis revealed the stratum corneum in the epidermis involved parakeratosis, infiltration of neutrophils and lymphocytes, and strong spongy edema. The dermis was infiltrated with lymphocytes, neutrophils, and a small number of eosinophils. Prednisolone was administered intravenously at 1-2 mg/kg/day for anti-inflammatory effect, but the effect was temporary. He required administration of broad-spectrum antibiotics for a long period, and he died at 121 days of life due to respiratory failure associated with pneumonia and sepsis. No consent for pathological autopsy was obtained from the parents.

**Supplementary Table S1**

| **Supplementary Table S1. List of primer sets used in PCR** | | |
| --- | --- | --- |
| Gene name |  | Sequence |
| **Genomic PCR & sequencing** | |  |
| h*ADAM17-*ex14 | Forward | 5´-ATGAGCACACACACAAACGC-3´ |
|  | Reverse | 5´-CCTGTGGCCTGTGAAGATGT-3´ |
| h*ADAM17-*ex15 | Forward | 5´-CCCTGAGGGGCACACTTTAG-3´ |
|  | Reverse | 5´-CAAAGCCTCCCACCACAGAA-3´ |
| **RT-PCR & sequencing** | |  |
| *hADAM17* | Forward | 5´-GTGATCCTGGCATCATGTATCTG-3´ |
|  | Reverse | 5´-CCATGCTGCTCAGCATTTCGACG-3´ |
| **qRT-PCR** |  |  |
| syn-h*ADAM17* | Forward | 5´-GATCTGACCGATCATCCTGTG-3´ |
|  | Reverse ^a^ | 5´-GTAATCCTCGAGGCATTCGG-3´ |
|  | Probe | 5´-AGAACCGCGTGGACAGCAAAGAAA-3´ |
| *Actb* (b-actin) | Forward | 5´-GGCTGTATTCCCCTCCATCG-3´ |
|  | Reverse | 5´-CCAGTTGGTAACAATGCCATGT-3´ |
| **Mutagenesis for syn-hADAM17** | |  |
| syn-hADAM17-C567R | Forward^b^ | 5´-CGCCTGGATCTGGGCAAATGCAAA-3´ |
|  | Reverse | 5´-CACGGTATCATCTTCGGCATTGCCA-3´ |
| syn-hADAM17-C600Y | Forward^c^ | 5´-ATAAAGTTTGCTGCCGCGATCTGTC-3´ |
|  | Reverse | 5´-AGCTATTATCGGTTTCGTTGCAGGCG-3´ |
| **Construction of expression plasmid for HiBiT-tagged syn-hADAM17** | | |
|  | Forward^d^ | 5´-GTGAGCGGCTGGCGGCTGTTCAAGAAGATTAGC GATGAAGGCGAAGAATGCGATCCTGGCATCATGTA-3´ |
|  | Reverse | 5´-CACACGGCTATTGCCGCAAACTTTGTTGCTGCGC TCTTGAAAGCACTC-3´ |
| ^a^Sequence of FLAG-tag | | |
| ^b^Mutated nucleotide sequence for syn-hADAM17-C567R was underlined. | | |
| ^c^Mutated nucleotide sequence for syn-hADAM17-C600Y was underlined. | | |
| ^d^Inserted nucleotide sequence for HiBit-tag was underlined. | | |

**Supplementary Table S2**

| **Supplementary Table S2. List of antibodies used in this study** | | |  |
| --- | --- | --- | --- |
| Antibody name | Vender^a^ | ID | Application^b^ |
| anti-FLAG | MBL | M185-3L | IHC (1:500 dilution)/ Western blotting |
| anti-ADAM17 | abcam | ab2051 | Western blotting |
| anti-GAPDH | Santa Cruz Biotechnology | sc-47724 | Western blotting |
| anti-rabbit IgG-HRP | Santa Cruz Biotechnology | sc-2004 | Western blotting |
| anti-mouse IgG-HRP | Santa Cruz Biotechnology | sc-2005 | Western blotting |
| anti-β-actin | Santa Cruz Biotechnology | sc-47778 | Western blotting |
| Alexa Fluor 488-labeled goat anti-mouse | Thermo Fisher Scientific | A11001 | IHC (1:500 dilution)/ |
| anti-ADAM17 | AdipoGen | D1(A12) | Blocking assay |
| ^a^abcam, Cambridge, UK; AdipoGen Life Sciences, San Diego, CA, USA; | | | |
| MBL, Medical & Biological Laboratories, Nagano, Japan; | | | |
| Santa Cruz Biotechnology, Santa Cruz, CA, USA; Thermo Fisher Scientific, Waltham, MA, USA. | | | |
| ^b^IHC, immunohistochemistry | | | |

**Supplementary Figure S1**

**
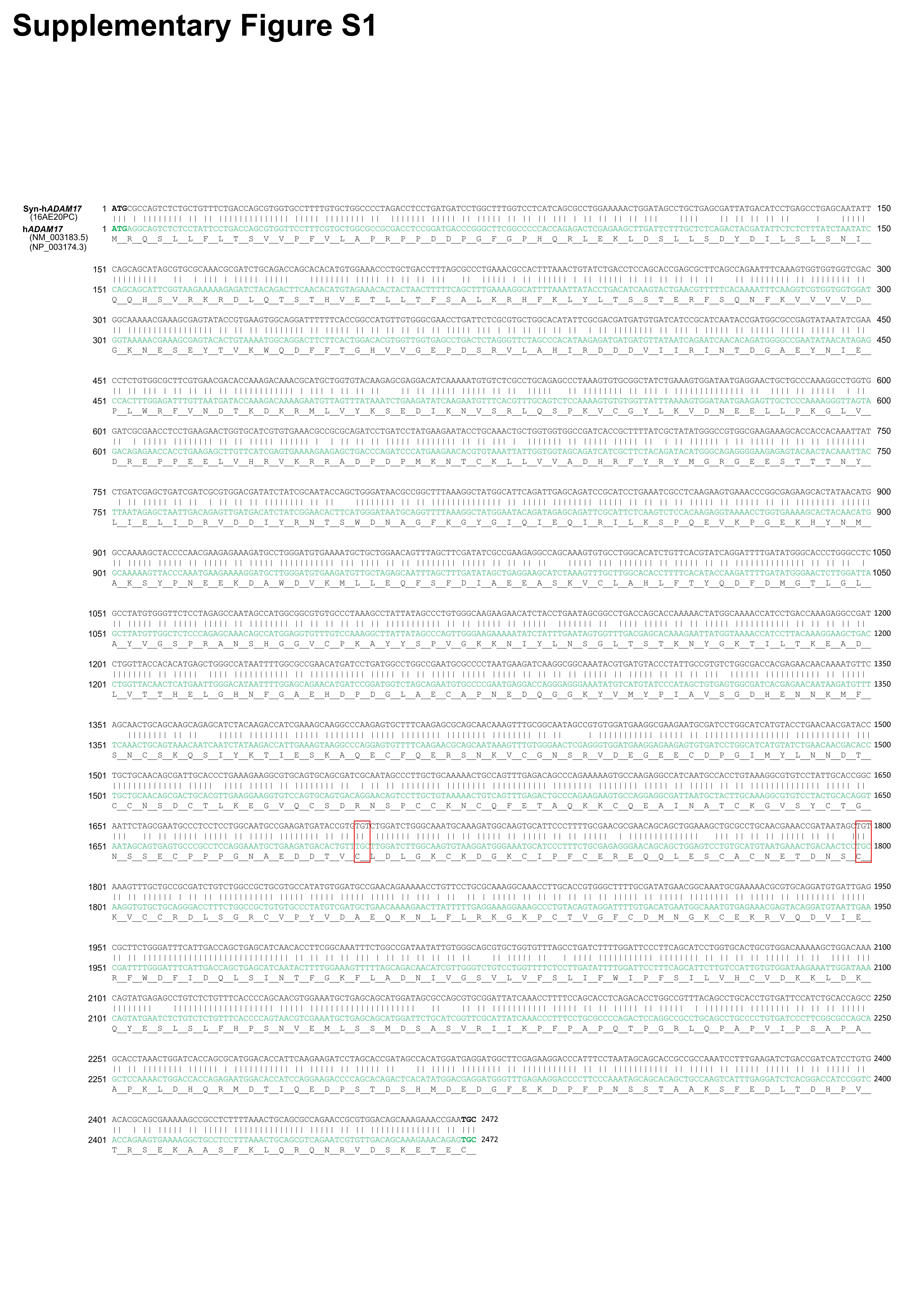
Supplementary Figure S1**

**Comparison of nucleic acid sequences of human a disintegrin and metalloprotease 17 (*ADAM17*) (NM_003183.5) and syn-hADAM17 used for functional analysis in this study.**

syn-hADAM17 is the artificial DNA fragment with optimised nucleotide sequence and encoding the same amino acid sequence as WT human ADAM17 (NM_003183.5). The DNA fragment was artificially synthesised and cloned into expression vectors. Red boxes indicate codons 567 and 600, whose amino acids were changed by single-base substitutions in the presented cases. Start and stop codons are shown in bold.

**Supplementary Figure S2**

**
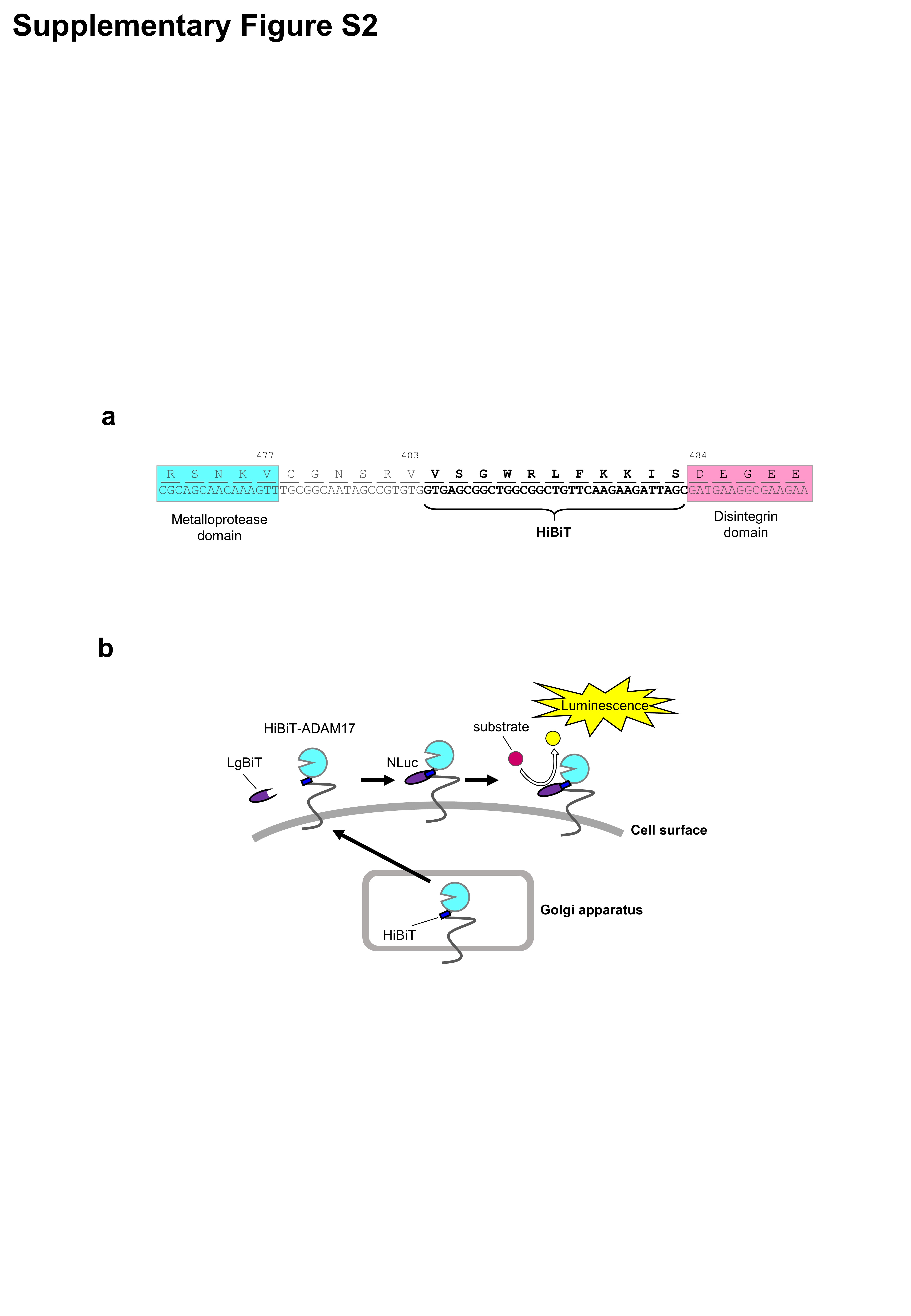
**

**Supplementary Figure S2**

**NanoBiT complementation assay to quantify** **HiBiT-tagged a disintegrin and metalloprotease 17 (ADAM17) expression on the cell surface.**

**(a)** Partial nucleotide sequence of expression plasmids of ADAM17 with the inserted HiBiT sequence (bold) between the metalloprotease domain (highlighted in blue) and disintegrin domain (highlighted in pink).

**(b)** Schematic of the use of NanoLuc Binary Technology (NanoBiT) complementation to detect cell-surface localisation of exogenously expressed HiBiT-tagged ADAM17. On addition of exogenous purified LgBiT (the large NanoLuc subunit) in the cell culture medium, complementation occurs between HiBiT and LgBiT to form the full-length NanoLuc (NLuc) luciferase on the cell surface. In the presence of the substrate furimazine, the complemented NLuc is luminescent.

**Supplementary Figure S3**

**
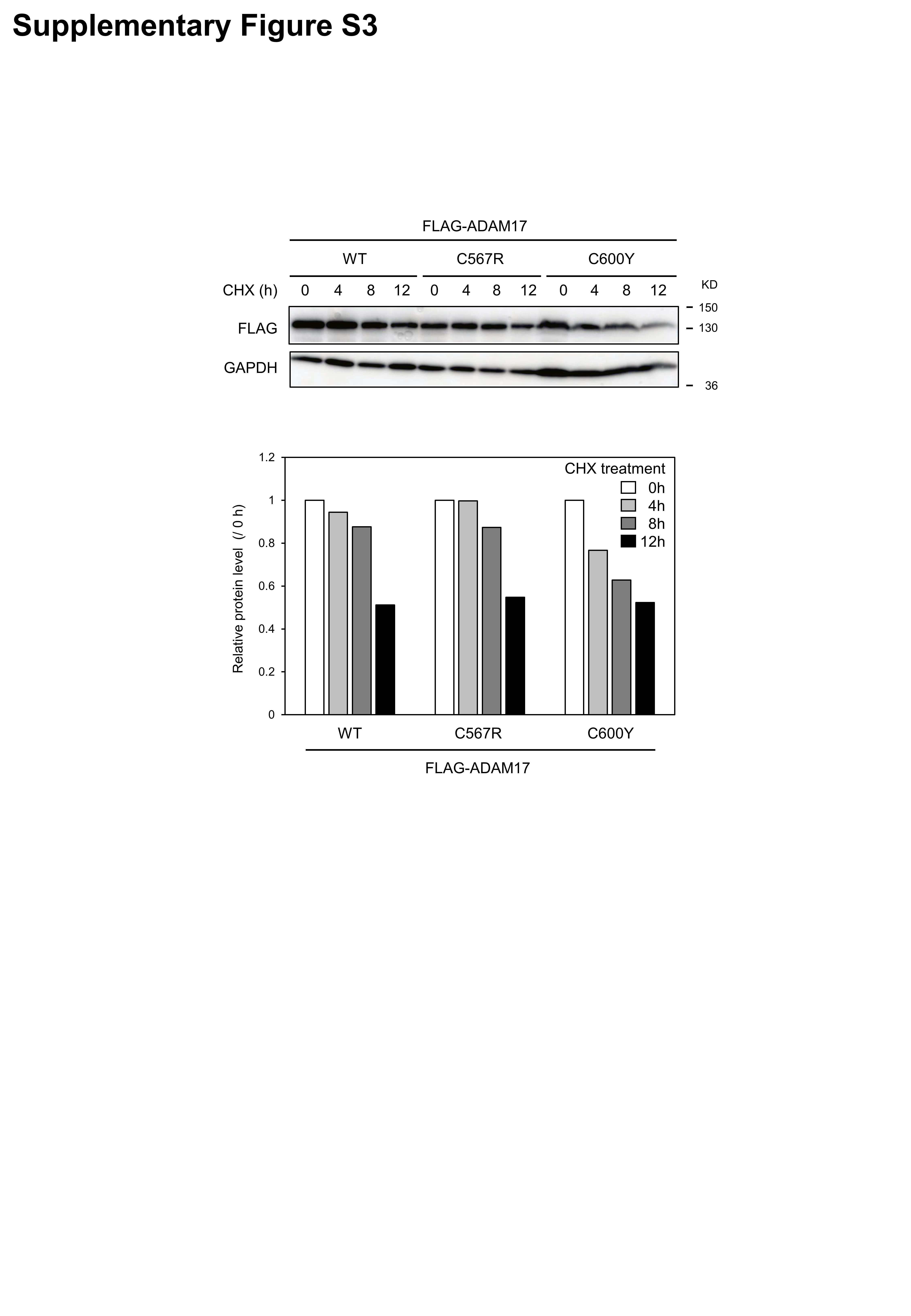
**

**Supplementary Figure S3**

**The effect of p.Cys567Arg and p.Cys600Tyr variants on the stability of a disintegrin and metalloprotease 17 (ADAM17).**

HEK293T cells transfected with pFLAG-syn-hADAM17 expression constructs and incubated for 48 h were exposed to 0.1 mg/mL cycloheximide (CHX) for indicated times. WT and mutant FLAG-syn-h ADAM17 were detected by western blotting using an anti-FLAG antibody, and intensities of specific bands corresponding to FLAG-tagged ADAM17 were quantified. Glyceraldehyde-3-phosphate dehydrogenase (GAPDH) was used as a loading control. Values are expressed as fold changes compared with those measured at time 0. The panel shows a representative result from three independent experiments with similar results. Full-length blots were presented in Supplementary Figure S5d.

**Supplementary Figure S4**

**
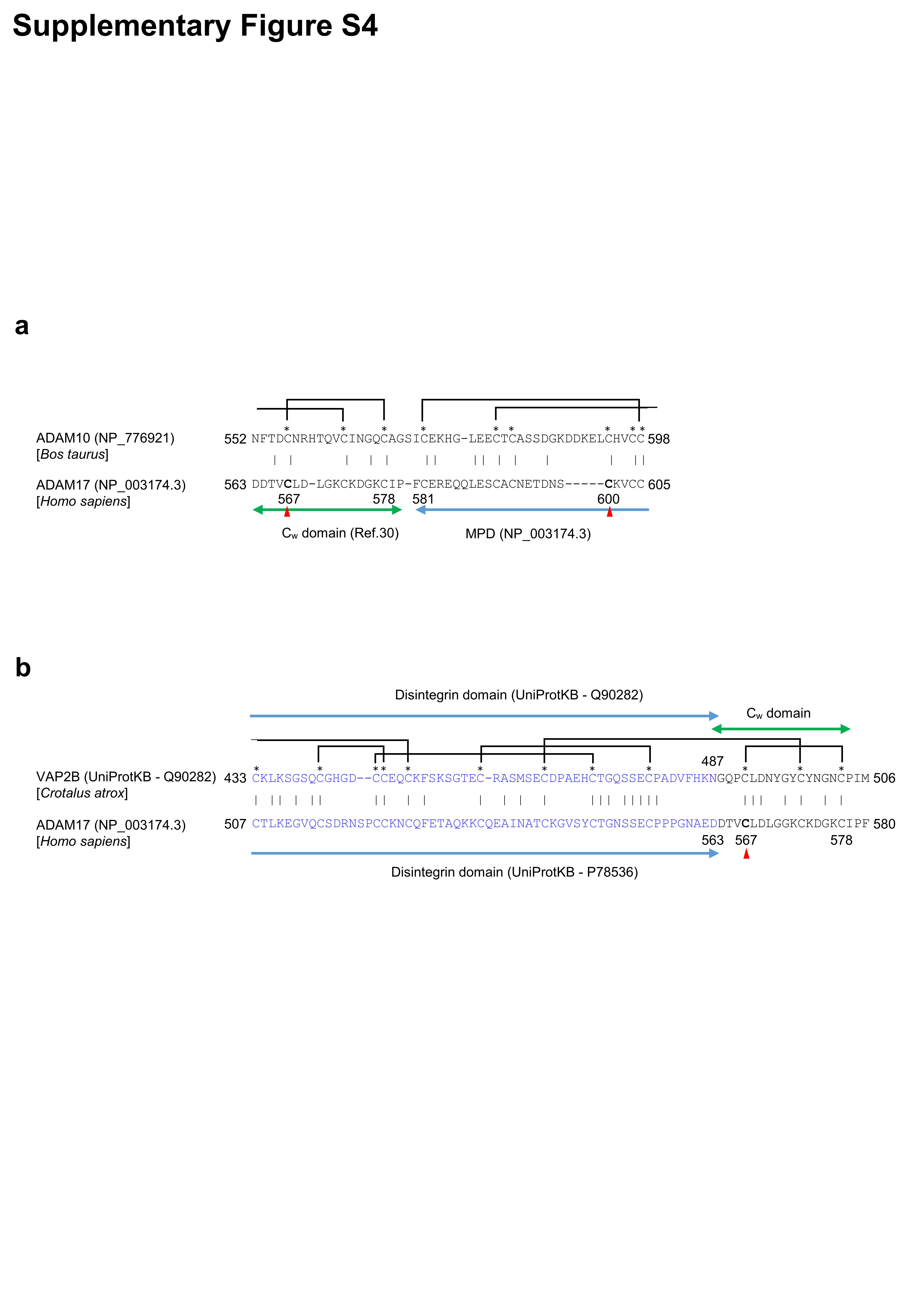
**

**Supplementary Figure S4**

**(a)** Sequence alignment of bovine a disintegrin and metalloprotease 10 (ADAM10) (NP_776921) and human ADAM17 (NP_003174.3) around variant codons detected in the presented cases. Disulfide bridges and domain structures (blue and green bars, respectively) are represented schematically.^29,30^ Conserved residues are indicated by vertical lines, and conserved cysteine residues are marked with asterisks. Two mutated amino acids (C567 and C600) detected in the presented cases are shown in bold and by red arrowheads.

**(b)** Sequence alignment of VAP2B (UniProtKB - Q90282), a metalloproteinase possessing a metalloproteinase/disintegrin/cysteine-rich domain architecture, from snake (*Crotalus atrox*) venom, and human ADAM17 (NP_003174.3) around their disintegrin domains.^34^ Disulfide bridges and domain structures (blue and green bars, respectively) are represented schematically.^34^ Conserved residues are indicated by vertical lines, and conserved cysteine residues are marked with asterisks. The mutated amino acid (C567) detected in the presented cases is shown in bold and by red arrowheads.

**Supplementary Figure S5**


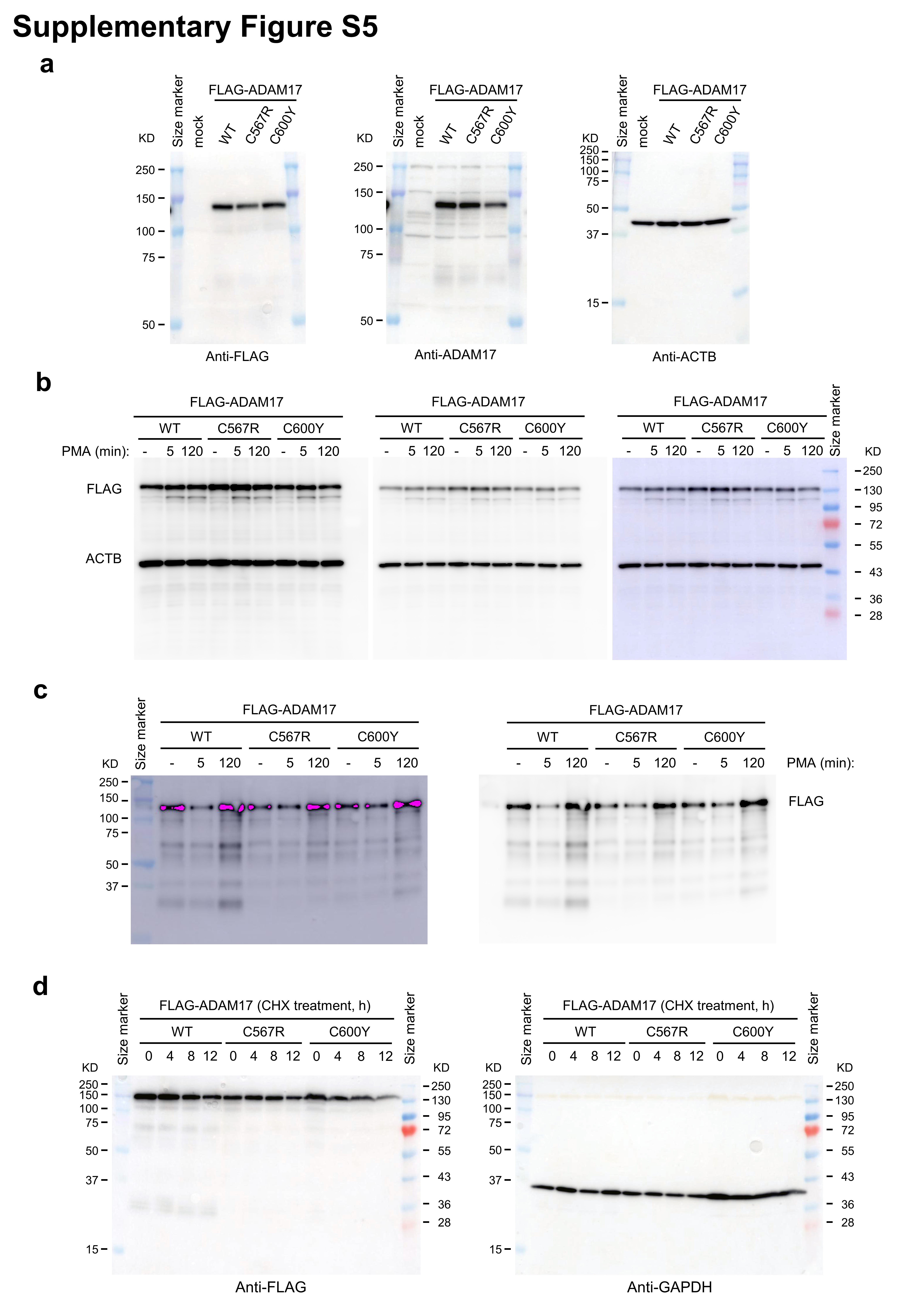


**Supplementary Figure S5**

**Full-length blots for preparing Figures and Supplementary Figure.**

**(a)** Blots used for cropped images in Figure 2b. Anti-FLAG (left), anti-ADAM17 (middle) and anti-ACTB (right) antibodies were used as the first antibodies for western blot analysis.

**(b)** Blots used for cropped images in Figure 3a. Blots with longer (left) and shorter (middle) exposures and blots with a size marker image (right) were included. Anti-FLAG and anti-ACTB antibodies were used as the first antibodies for western blot analysis.

**(c)** Blots with (left) and without (right) a size marker image used for Figure 3b. Anti-FLAG antibody was used as the first antibody for western blot analysis. In the exposure condition for a blot with size marker, excess signals were detected from bands of FLAG-tagged ADAM17 proteins

**(d)** Blots for cropped images in Supplementary Figure S3 with size marker images. Anti FLAG (left) and anti-GAPDH (right) antibodies were used as the first antibodies for western blot analysis.
